# Step-Wise Loss of Bacterial Flagellar Torsion Confers Progressive Phagocytic Evasion

**DOI:** 10.1371/journal.ppat.1002253

**Published:** 2011-09-15

**Authors:** Rustin R. Lovewell, Ryan M. Collins, Julie L. Acker, George A. O'Toole, Matthew J. Wargo, Brent Berwin

**Affiliations:** 1 Department of Microbiology and Immunology, Dartmouth Medical School, Lebanon, New Hampshire, United States of America; 2 Department of Microbiology and Molecular Genetics, University of Vermont College of Medicine, Burlington, Vermont, United States of America; Yale University School of Medicine, United States of America

## Abstract

Phagocytosis of bacteria by innate immune cells is a primary method of bacterial clearance during infection. However, the mechanisms by which the host cell recognizes bacteria and consequentially initiates phagocytosis are largely unclear. Previous studies of the bacterium *Pseudomonas aeruginosa* have indicated that bacterial flagella and flagellar motility play an important role in colonization of the host and, importantly, that loss of flagellar motility enables phagocytic evasion. Here we use molecular, cellular, and genetic methods to provide the first formal evidence that phagocytic cells recognize bacterial motility rather than flagella and initiate phagocytosis in response to this motility. We demonstrate that deletion of genes coding for the flagellar stator complex, which results in non-swimming bacteria that retain an initial flagellar structure, confers resistance to phagocytic binding and ingestion in several species of the gamma proteobacterial group of Gram-negative bacteria, indicative of a shared strategy for phagocytic evasion. Furthermore, we show for the first time that susceptibility to phagocytosis in swimming bacteria is proportional to *mot* gene function and, consequently, flagellar rotation since complementary genetically- and biochemically-modulated incremental decreases in flagellar motility result in corresponding and proportional phagocytic evasion. These findings identify that phagocytic cells respond to flagellar movement, which represents a novel mechanism for non-opsonized phagocytic recognition of pathogenic bacteria.

## Author Summary

Flagella-driven bacterial motility, referred to as swimming, has been recognized for over 20 years to affect the ability of bacteria to infect and colonize a host. The common theme is that bacteria must be motile to colonize the host but must become non-motile to chronically persist; this has been observed in many pathogenic bacteria including species of *Vibrio* and *Pseudomonas*. Therefore it makes sense that the immune system would evolve mechanisms to exploit this virulence determinant of pathogenic bacteria. Here we present evidence that flagellar motility is recognized by innate immune cells as a phagocytic activation signal. We show that step-wise loss of flagellar motility confers a proportional ability to evade phagocytic engulfment, independent of the flagellum itself acting as a phagocytic activator. This is not due to motility- co-regulated secretions or compensatory genetic changes by the bacteria, but instead is due to a mechano-sensory response whereby phagocytic cells respond directly to flagellar motility. This represents a novel mechanism by which the innate immune system facilitates clearance of bacterial pathogens, and provides an explanation for how selective pressure may result in bacteria with down-regulated flagellar gene expression and motility as is observed in isolates taken from chronic infections.

## Introduction

Pathogen recognition by the innate immune system is one of the first lines of defense in cellular immunity to infection [1]. However, how bacteria establish chronic infections, as observed in patients with cystic fibrosis (CF), and the reasons that these infective agents cannot be eliminated by the immune system are still largely unclear [2], [3]. A relevant example of this is *Pseudomonas aeruginosa*, a Gram-negative opportunistic pathogen which establishes infection in the lung tissue of CF patients and effectively evades immune clearance [2], [3]; CF disease severity correlates with chronic infection of the pulmonary compartment by *P. aeruginosa* [2], [3]. One contributing factor that enables immune evasion is the loss of bacterial flagellar motility during colonization [4]–[8]. *P. aeruginosa* has a single, polar, monotrichous flagellum which provides force for swimming locomotion in aqueous environments [9]. Multiple studies have found that the majority of *P. aeruginosa* isolates taken from chronically infected CF patients have down-regulated flagellar gene expression and are phenotypically deficient in the ability to swim [6], [7]. The previous paradigm suggested that the loss of flagellin as a phagocytic ligand facilitates evasion of innate immune cells and results in increased bacterial burden in the CF lung [5], [8]. Recently, with the use of flagellated and non-flagellated swimming-defective *P. aeruginosa* genetic mutants, we demonstrated that it is not the loss of the flagellum itself, but rather the loss of flagellar-based swimming motility that allows *P. aeruginosa* to avoid phagocytic clearance [4]. However, it is currently unclear how the loss of bacterial swimming motility enables phagocytic evasion from innate immune cells and, to date, no published reports have examined in detail the dynamics of non-opsonized *P. aeruginosa*-phagocyte association and subsequent fate as a function of bacterial swimming motility.

In order to delineate how bacterial swimming contributes to phagocytic recognition and uptake, we take advantage of isogenic bacterial mutations that affect flagellar swimming motility and we identify the individual components that comprise the phagocytic process as it relates to swimming and non-swimming bacteria. Swimming motility in Gram-negative bacteria is powered by generation of an ion gradient to turn a flagellar rotor against a stationary stator complex [10]. The resultant force provides the necessary torque to turn the flagellar filament and thus propel the bacteria [10]. In these studies we utilize genetic mutants which lack structural and functional flagella due to mutations in either the flagellin monomer or the flagellar hook protein and are therefore non-swimming, and also mutants which do not produce all or part of the flagellar stator complex. These *mot* stator mutants all have fully assembled flagella, since loss of the Mot stator proteins does not impede construction of the flagellar filament, and are instead partially or fully defective in the ability to rotate the flagellum depending on which stator components are omitted [9], [11], [12]. Our previous work with these mutants found that the phagocytic response to *P. aeruginosa* infection depends on flagellar motility, but does not depend on the flagellum itself as an activating ligand [4].

Since loss of flagellar motility confers phagocytic resistance, these data suggest that innate immune cells have the ability to recognize bacterial movement and that swimming bacteria provide an important sensory input for phagocytic engulfment [4]. However, an alternative explanation is that bacteria change the expression of unknown secreted and/or cell-surface ligands in response to the loss of swimming motility and therefore alter their phagocytic recognition and uptake. Here we test these hypotheses and provide the first evidence that phagocytic cells utilize bacterial swimming motility as a global mechanism for bacterial recognition. Significantly, we show that alterations in swimming motility allow multiple bacterial species to evade phagocytic recognition. This is not due to measurable changes in the expression of common outer membrane proteins (OMPs) or known regulators of pathogen-associated molecular patterns (PAMPs). Rather, we provide evidence that phagocytic cells are able to respond to bacterial swimming as a function of flagellar rotation after initial contact and, importantly, that phagocytosis is directly proportional to the flagellar torque of the bacteria. We therefore propose a model in which the step-wise loss of flagellar function confers a progressive increase in the ability of the bacteria to evade the phagocytic response of the innate immune system, which promotes an environmentally beneficial niche during infection. This selective pressure provides an explanation for the down-regulation of motility genes and phenotypic loss of swimming that is observed in isolates procured from chronic infections [4]–[8].

## Results

### Loss of flagellar motility is a widespread mechanism amongst Gram-negative bacteria for resistance to phagocytic uptake

To determine whether phagocytic evasion through loss of swimming motility is specific to *P. aeruginosa* or is a mechanism shared amongst flagellated Gram-negative pathogens, we used genetically modified motility mutants in multiple bacterial backgrounds (Table 1). *P. aeruginosa* PA14 is a non-mucoid clinical isolate and is considered the wild-type (WT) in this study. All *P. aeruginosa* genetic mutants used in this study are on the PA14 background. All *Vibrio cholerae* mutants are constructed using the classical biotype O395 strain and all *Escherichia coli* mutants are in the K12 background. All non-flagellated strains (which lack swimming motility) have a mutation in either the flagellar hook gene (*flgK*), or in the gene coding for the flagellin monomer (*flaA* and *fliC* for *V. cholerae* and *E. coli*, respectively) [9], [12], [13].

**Table 1:** Bacterial strains used in this study.

Strain	Genotype/Description	Phenotype	Reference or Source
*P. aeruginosa* FRD1	mucoid clinical isolate	Fully assembled flagellum, swimming competent	13
*P. aeruginosa* PA14	non-mucoid clinical isolate, WT	Fully assembled flagellum, swimming competent	9
*flgK*	*flgK*::*Tn5* flagellar hook protein	No flagellum, non-swimming	9
*ΔmotAB*	*ΔmotAB* flagellar stator proteins	Fully assembled flagellum, swimming competent	9
*ΔmotCD*	*ΔmotCD* flagellar stator proteins	Fully assembled flagellum, swimming competent	9
*ΔmotABΔmotCD*	*ΔmotAB ΔmotCD* flagellar stator proteins	Fully assembled flagellum, non-swimming	9
*E. coli* K12	WT	Fully assembled flagella, swimming competent	*E. coli* Genetic Stock Center
*ΔflgK*	*ΔflgK* flagellar hook protein	No flagella, non-swimming	*E. coli* Genetic Stock Center
*ΔfliC*	*ΔfliC* flagellin monomer	No flagella, non-swimming	*E. coli* Genetic Stock Center
*ΔmotA*	*ΔmotA* flagellar stator protein	Fully assembled flagella, non-swimming	*E. coli* Genetic Stock Center
*V. cholerae* O395	WT	Fully assembled flagellum, swimming competent	12
*ΔflaA*	*ΔflaA* flagellin monomer	No flagellum, non-swimming	12
*ΔmotX*	*ΔmotX* flagellar stator protein	Fully assembled flagellum, non-swimming	12
*ΔtcpA*	*ΔtcpA* toxin co-regulated pilin monomer	Fully assembled flagellum, swimming competent, lacks TCP	18
*ΔtoxT*	*ΔtoxT* toxin co-regulated pili regulator protein	Fully assembled flagellum, swimming competent, lacks TCP	17

**Figure 1:**
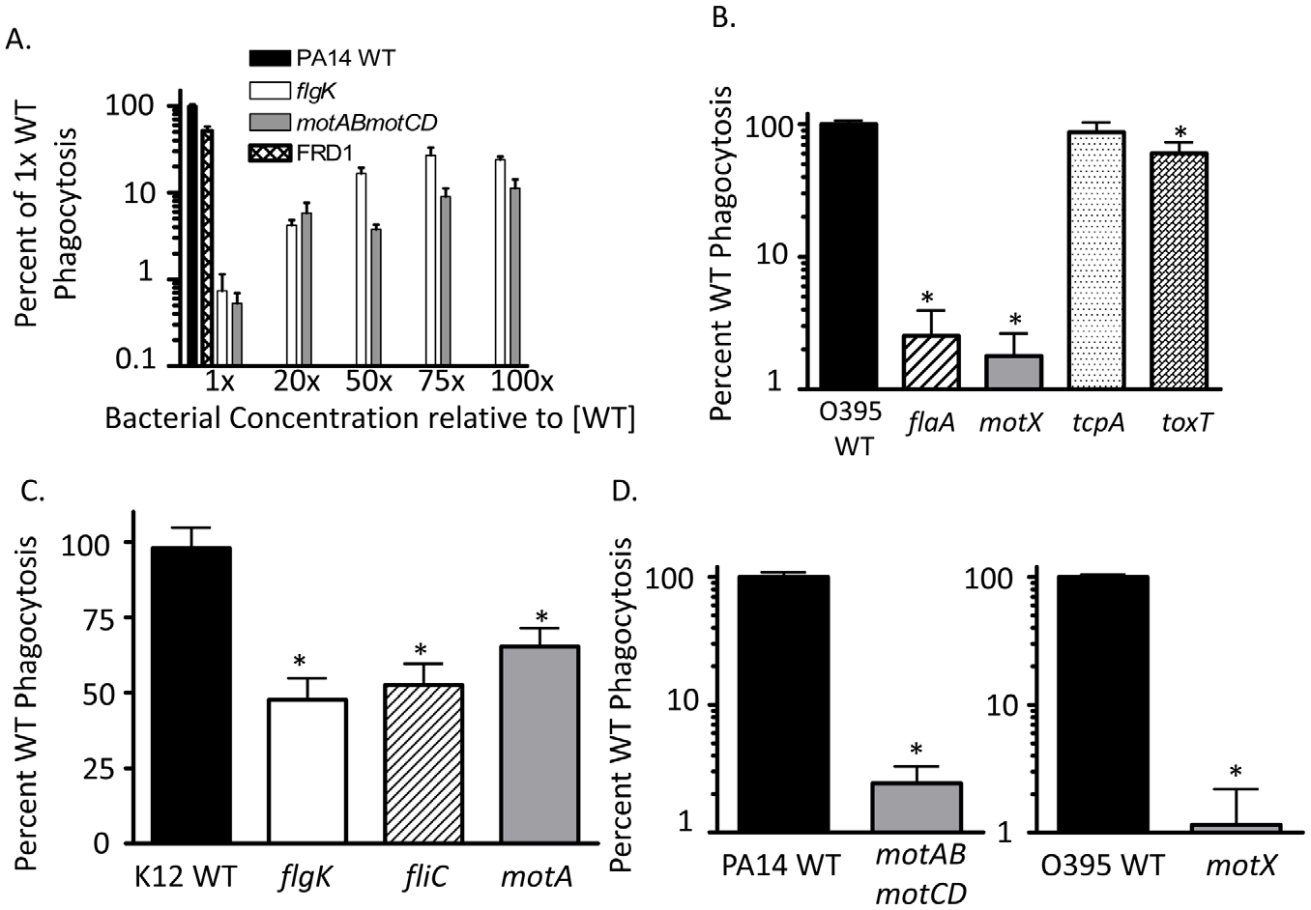
Non-swimming gram-negative bacteria are resistant to phagocytosis. Gentamicin protection assays were used to assess: (A) C57BL/6 BMDC phagocytosis of WT *P. aeruginosa* strain PA14, the independent mucoid clinical isolate FRD1, and increasing concentrations of non-flagellated mutant *flgK* and flagellated but non-swimming mutant *motABmotCD* (in PA14 background). (B) Murine BMDC phagocytosis of WT *V. cholerae* strain O395, and the *flaA, motX, tcpA,* and *toxT* mutants. (C) Murine BMDC phagocytosis of WT *E. coli* strain K12, and the *flgK, fliC,* and *motA* mutants. (D) Human THP-1 leukocyte phagocytosis of *P. aeruginosa* WT, *flgK,* and *motABmotCD* (left); or *V. cholerae* WT, *flaA,* and *motX* (right). Where indicated throughout this and the other figures, phagocytosis of WT strains (PA14, K12 and 0395 in this figure) has been normalized to 100% and the relative phagocytosis of the mutant strains shown as the percent of WT. N≥6, *p<0.05 compared to WT.

The two stator complexes (MotAB and MotCD) in *P. aeruginosa* are each composed of two proteins and are functionally partially-redundant. Importantly, deletion of all four genes (*motABmotCD*) inhibits flagellar rotation, but not flagellar assembly, resulting in a mutant that is flagellated but incapable of swimming [9]. The *motAB* mutant, and to a lesser extent the *motCD* mutant, are swimming competent, though not to the same degree as the parental WT [9], [14]. The stator complexes in *V. cholerae* and *E. coli* are analogous to those of *P. aeruginosa*, though not identical in composition. The stator of *V. cholerae* is also composed of at least four proteins, termed PomA, PomB, MotX, and MotY [15]. The contribution of each protein to stator functionality in *V. cholerae* is still unclear, however loss of the *motX* gene results in a flagellated, but non-swimming mutant that is phenotypically similar to the *P. aeruginosa motABmotCD* mutant [12], [15]. In *E. coli*, the stator is composed of only two proteins, MotA and MotB [13]. Loss of either gene product (MotA in this study) results in a similar flagellated, but non-swimming mutant [13]. We previously reported that the genetic loss of the stator complexes in *P. aeruginosa* PA14 confers resistance to phagocytosis *in vitro* and *in vivo* in comparison to the swimming-competent parental strain [4]. Phagocytic evasion is not dependent on flagellar assembly, as both flagellated and non-flagellated mutants were equally capable of avoiding phagocytic ingestion [4]. In order to better understand the dynamics of phagocytic resistance by strains incapable of swimming motility, we first verified that strains competent in swimming motility were as equally susceptible to gentamicin as non-swimming strains and remained equally viable during incubation (Figure S1 and data not shown), and then performed gentamicin protection assays with bone marrow-derived dendritic cells (BMDCs) and increasing concentrations of non-swimming *P. aeruginosa* relative to the WT concentration. We were not able to identify a resistance threshold in either the *flgK* or the *motABmotCD* mutants where phagocytic susceptibility approximated WT levels (Figure 1A). In assays where the concentration of non-swimming bacteria was increased to 100-times that of WT, we observed only a ∼30% increase in recovery relative to WT (Figure 1A), indicating that the mechanism facilitating phagocytic resistance of non-swimming *P. aeruginosa* can only partially be overcome even in the presence of increased non-swimming bacterial concentrations. This degree of phagocytic resistance conferred by loss of bacterial motility is highlighted by the comparison to other phenotypes that have been reported to alter bacterial clearance. For example, alginate production (mucoidy) by *P. aeruginosa* has been reported to alter bacterial phagocytic susceptibility [16], however the swimming mucoid *P. aeruginosa* strain FRD1 [17] exhibited only a ∼2-fold change in phagocytosis compared to non-mucoid PA14 WT (Figure 1A).

To test whether motility-based phagocytic recognition is specific to *P. aeruginosa*, or if this mechanism extends to other bacterial pathogens as well, we performed similar assays using flagellated and non-flagellated *V. cholerae* and *E. coli* genetic mutants that contain analogous mutations to the *P. aeruginosa* mutants described previously. In assays using *V. cholerae*, both the non-flagellated *flaA* mutant and the flagellated but non-swimming *motX* mutant were ∼100-fold more resistant to phagocytosis than the isogenic WT (Figure 1B). In comparison, the swimming-competent *tcpA* and *toxT* mutants, which instead lack toxin co-regulated pili (TCP) which facilitate attachment [18]–[20], were ingested to a similar degree as WT *V. cholerae* (Figure 1B). In experiments using *E. coli*, the non-flagellated *flgK* and *fliC* strains and the flagellated but non-swimming *motA* strain were all significantly more resistant to phagocytosis compared to the swimming WT, although to a lesser degree than observed with *P. aeruginosa* and *V. cholerae* (Figure 1C). To test if these findings also applied to human phagocytes, we tested human THP-1 cells for their preferential ability to phagocytose swimming bacteria. The human THP-1 phagocytic cell line recapitulated our observations using murine BMDCs (Figure 1D) which supports a general mechanism by which non-opsonized Gram-negative bacterial recognition by phagocytic cells is swimming motility-dependent and is not species-specific.

### *P. aeruginosa* lacking swimming motility have decreased overall association with innate immune cells independent of flagellar assembly

**Figure 2:**
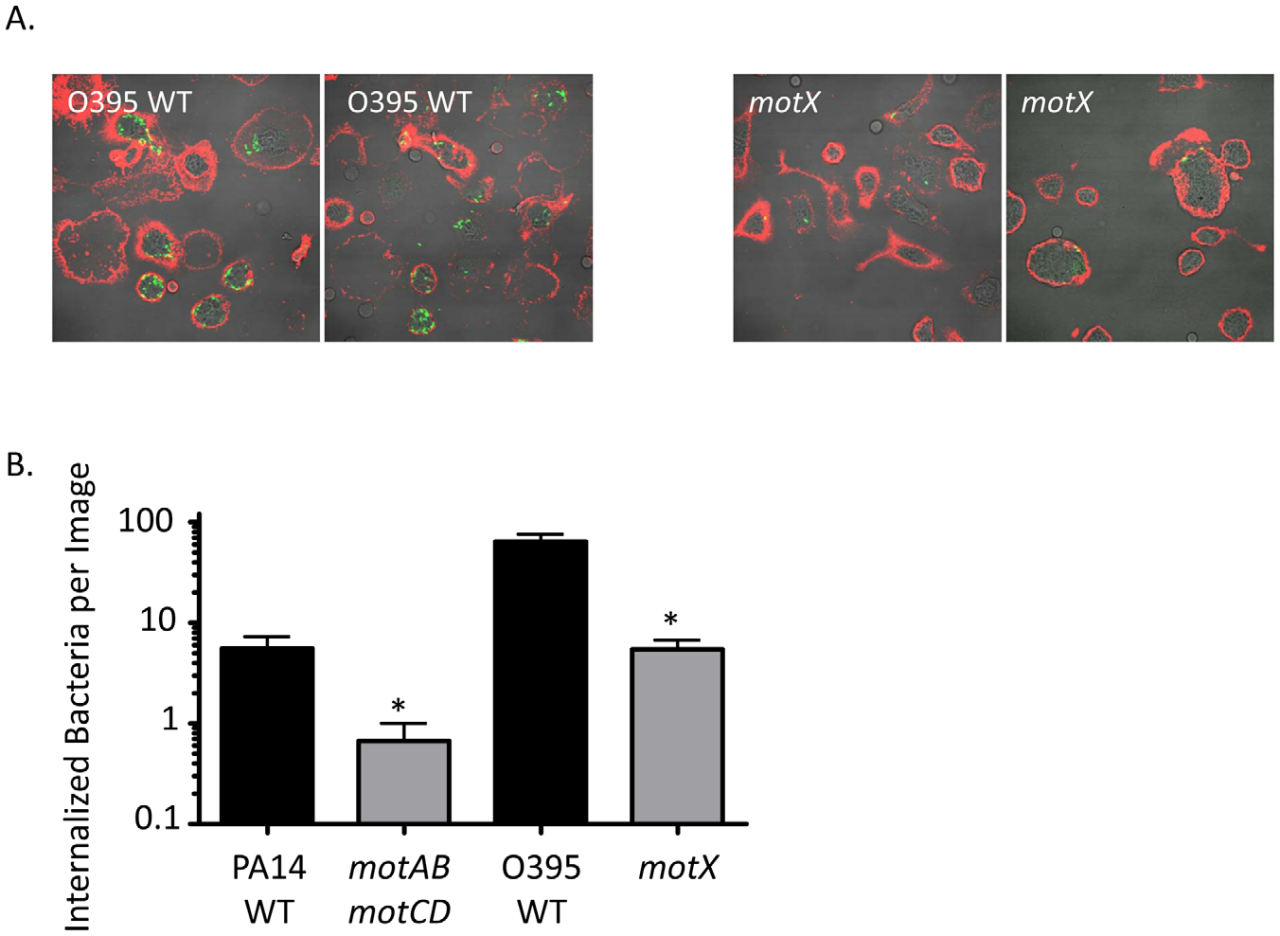
Fluorescence microscopy of phagocytic interactions with GFP-expressing bacteria. (A) Confocal fluorescence microscopy of untreated murine peritoneal macrophages co-incubated at 37°C for 45 minutes with GFP-transformed *V. cholerae* O395 WT (left) or *motX* (right), washed, and subsequently stained on ice with Alexa647-conjugated wheat germ agglutinin (WGA). (B) Internalized bacteria, as in (A), were quantified on the basis of being within a contiguous WGA-decorated phagocyte plasma membrane and not co-localizing with WGA (co-localization seen as yellow, as at the plasma membrane or being external to a phagocytic cell). N≥6 images, *p<0.05.

In order to visualize the host-pathogen interactions that occur between *P. aeruginosa* and innate immune cells, and to confirm the assays presented in Figure 1, murine peritoneal macrophages were incubated at 37°C with equal numbers of either GFP-transformed *P. aeruginosa* PA14 WT or *motABmotCD*, or *V. cholerae* O395 WT or *motX* bacteria and the non-adherent bacteria were washed away prior to counter-staining exposed cell-surfaces with Alexa-647-labeled wheat germ agglutinin (WGA). Multiple images per co-incubation were generated by randomly choosing a viewing field and counting the internalized bacteria along the Z-axis of all visible cells. Representative images of co-incubations using O395 WT (Figure 2A, left) or *motX* (Figure 2A, right) demonstrate that bacteria with swimming motility associate with macrophages to a much higher extent than do non-swimming bacteria. In co-incubations using O395 WT or PA14 WT bacteria (as in Figure 2A) the quantified internalization, as assessed by bacteria within the phagocytes that do not co-localize with the WGA, is increased >10-fold over *motX* or *motABmotC*D, respectively (Figure 2B). These data both further support our gentamicin protection assays and the hypothesis that loss of flagellar motility inhibits the ability of phagocytic cells to engulf bacteria.

### Increased phagocytic resistance by *P. aeruginosa motABmotCD* is not due to compensatory changes in bacterial secretions, extracellular protein expression, or PAMP presentation

**Figure 3:**
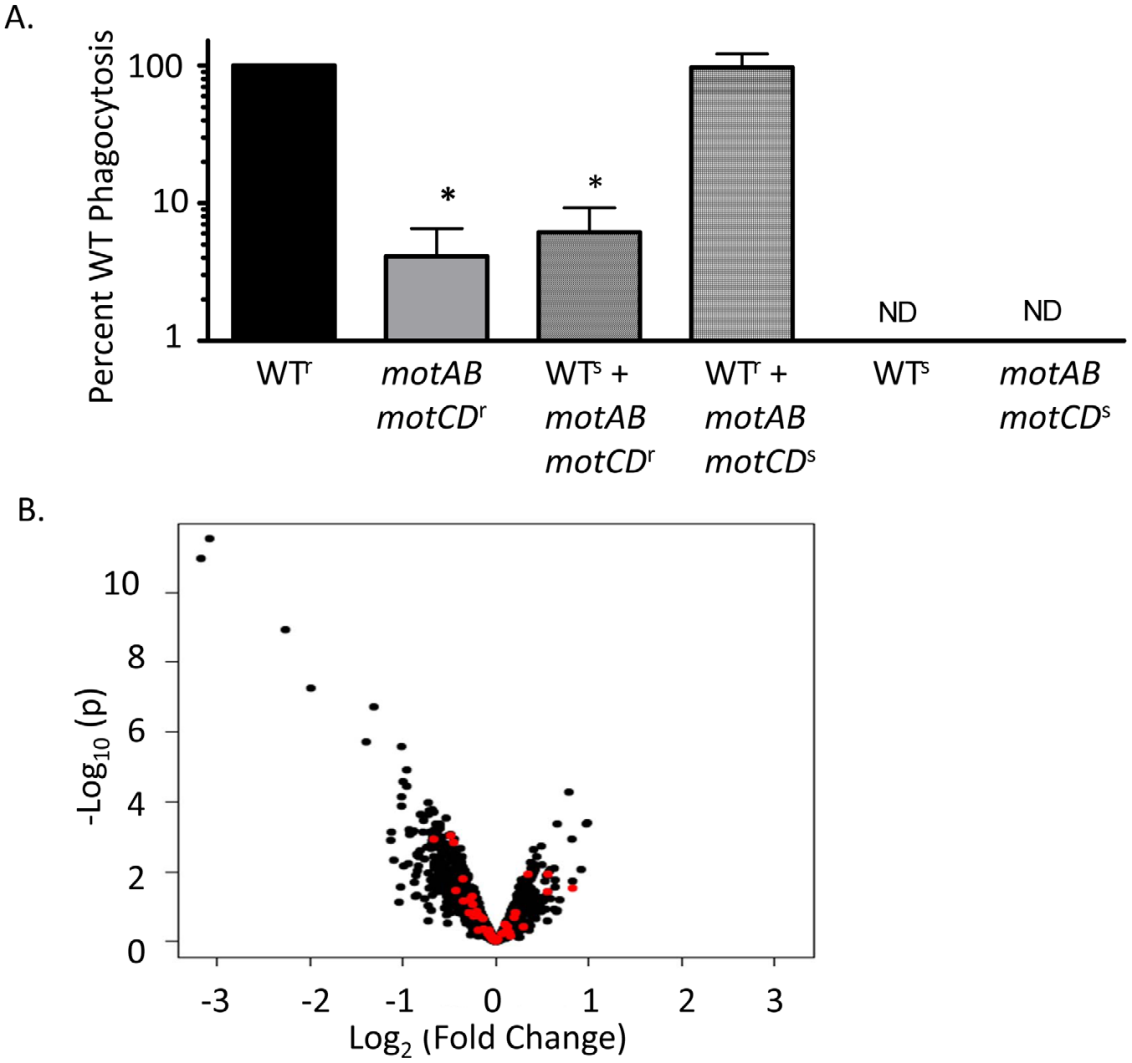
The phagocytic resistance by *P. aeruginosa motABmotCD* is not due to resultant changes in bacterial secretions, extracellular protein expression, or PAMP regulation. (A) BMDCs were co-incubated with a mixture of equal numbers of carbinicillin-resistant PA14 WT and carb-sensitive *motABmotCD* or, conversely, carb-resistant *motABmotCD* and carb-sensitive WT. Phagocytic susceptibility was assayed by gentamicin protection assay and plating on carbinicillin-treated LB agar. (B) Volcano plot of WT gene expression versus *motABmotCD* mutant gene expression. Red points indicate genes corresponding to likely immunogenic molecules (see Table S1). N≥7, *p<0.05.

One possible explanation for our current observations is that motility or loss of motility elicits the release of an unknown soluble factor, and that this hypothetical ligand is acting to either induce phagocytosis (if elicited in the motile bacteria) or to impair phagocytosis (if elicited in the non-motile bacteria) by affecting either the neighboring bacteria or the phagocyte itself. In either scenario, we hypothesized that one bacterial strain may affect the phagocytosis of the other strain *in trans*. We tested this hypothesis with mixed cultures of PA14 WT and *motABmotCD.* Carbinicillin-resistant (Carb^r^) WT or the *motABmotCD* mutant were mixed in equal numbers with the Carbinicillin-sensitive (Carb^s^) version of the other strain and introduced to murine BMDCs in a standard gentamicin protection assay, after which lysates were plated on Carbinicillin-selective plates. The number of recovered Carb^r^-*motABmotCD* CFUs after co-incubation with Carb^s^-WT and BMDCs was not significantly different than when *motABmotCD* alone was incubated with BMDCs (Figure 3A). Likewise, recovered CFUs of Carb^r^-WT when mixed with Carb^s^-*motABmotCD* did not change from what is observed when WT alone is assayed by gentamicin protection assay (Figure 3A). This indicates that a swimming competent strain is not able to confer phagocytic susceptibility to a non-swimming strain, nor can a non-swimming mutant confer resistance to a swimming WT. Therefore, differences in phagocytic response as elicited by swimming verses non-swimming *P. aeruginosa* are not due to any soluble factor being secreted into the extracellular environment or altering the phagocytic activity of the BMDCs.

Many of the regulatory pathways controlling synthesis of outer membrane proteins and peripheral structures on *P. aeruginosa* are still being elucidated; however phagocytosis assays with *P. aeruginosa* swarming mutants, type-III secretion mutants, and mucoid strains did not result in significantly increased phagocytic resistance relative to controls (data not shown). Nonetheless, it is still possible that flagellar rotation is co-regulated with gain or loss of expression of an unknown extracellular PAMP or ligand that is recognized by innate immune cells. To identify if deletion of the *mot* genes correlates with changes in peripheral gene expression levels, we performed genome-wide microarray analysis of the WT and *motABmotCD* strains. Comparison of gene expression levels between WT and *motABmotCD* showed no significant change in any recognizable PAMP regulators, OMP genes, lipopolysaccharide synthesis elements, or known immune activating factors (Figure 3B and Table S1). Genes which did change expression more than 2-fold with loss of the *mot* operons are listed in Table 2. However, swimming motility assays and preliminary phagocytic assays with PA14 strains containing transposon insertions in each of those genes identified in Table 2 did not recapitulate the phenotypes observed with *motABmotCD* (data not shown). These data support the hypothesis that phagocytic cells are able to directly respond to swimming motility by bacteria.

**Table 2:** Change in gene expression ≥2-fold with loss of *motABmotCD*.

Gene ID	Product name	*motABmotCD-*WT log(Fold Change)	False Discovery Rate
PA0122	hypothetical protein	−0.941469596	0.007242301
PA0179	hypothetical protein	−2.006827619	8.04E-05
PA1494	hypothetical protein	0.637413177	0.047892072
PA2171	hypothetical protein	0.766622785	0.021035671
PA2462	hypothetical protein	−0.66144003	0.045332067
PA3126	IbpA	−0.934812048	0.042331228
PA3385	AmrZ	−2.266352363	6.60E-07
PA3496	hypothetical protein	−0.999680884	0.047892072
PA3662	hypothetical protein	−3.149905237	7.25E-09
PA3740	hypothetical protein	−1.34733663	0.000845588
PA4033	hypothetical protein	−1.013138014	0.015126456
PA4387	hypothetical protein	−0.806309344	0.047892072
PA4683	hypothetical protein	−1.389032018	0.000518982
PA4843	hypothetical protein	−3.123519763	6.85E-10
PA4953t	MotB	−0.69302142	0.014485643
PA5053	HslV	−0.732046986	0.049682967
PA5446	hypothetical protein	−1.010684502	0.01227902

### Inherent microbiocidal activity and limited bacteria-cell contact does not provide for phagocytic resistance in non-swimming bacteria

**Figure 4:**
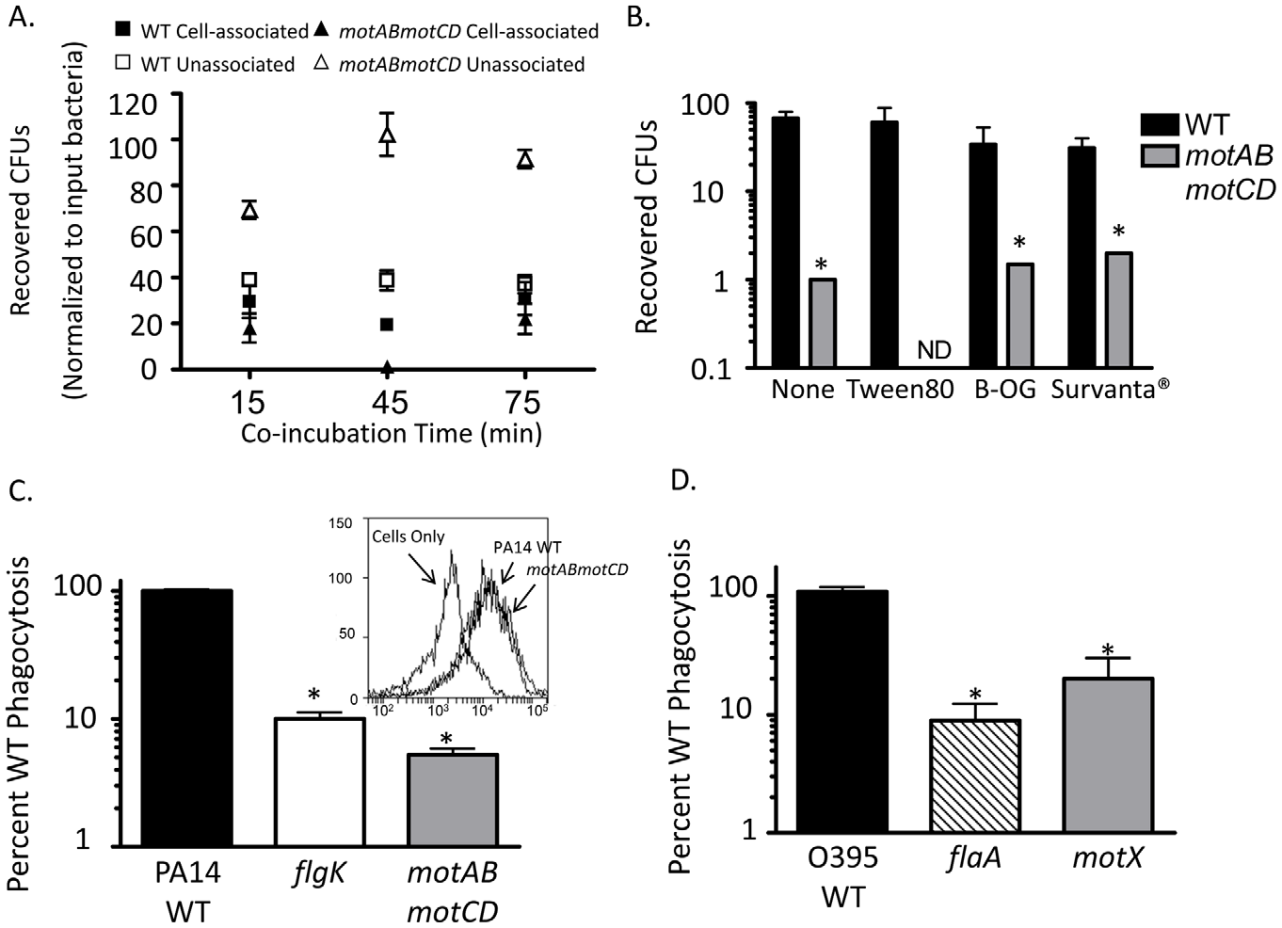
Microbiocidal activity and limited bacteria-cell contact does not provide for decreased phagocytic clearance of non-swimming bacteria. (A) Adherent murine peritoneal macrophages were co-incubated with PA14 WT or *motABmotCD* and cellular associated bacteria and non-associated bacteria were quantitatively assessed at the indicated time points. (B) BMDCs were co-incubated with WT or *motABmotCD* in the presence or absence of 0.01% Tween80, 0.01% beta-octyl glucoside, or 2% Survanta, and assayed by gentamicin protection assay for relative bacterial phagocytosis. (C, inset) GFP-expressing PA14 WT or *motABmotCD* were centrifuged onto BMDCs and immediately fixed and analyzed by FACs for cellular association. Phagocytic cells in the absence of bacteria are shown as background. (C) *P. aeruginosa* PA14 WT, *flgK*, or *motABmotCD*, or (D) *V. cholerae* O395 WT, *flaA*, or *motX* were centrifuged onto BMDCs or peritoneal macrophages, respectively, and assayed by gentamicin protection assay. N≥5, *p<0.05 as compared to WT.

An alternative hypothesis to the cellular sensing of bacterial motility is that instead of non-swimming strains evading phagocytic uptake, the loss of flagellar motility renders the bacteria more susceptible to killing within the phagolysosome. While there is no prior evidence of this, we rigorously tested relative bacterial association and recovery over time by co-incubating WT or *motABmotCD* with adherent macrophages and then separating the cell-unassociated bacteria in the media from the macrophage-associated bacteria and plating both fractions to quantitatively assess relative CFUs in each. At all time points tested, greater CFU recovery was observed in the unassociated fraction when using *motABmotCD*, while in the associated fraction, significantly higher CFUs were recovered with WT (Figure 4A). If intracellular killing were increased for *motABmotCD*, extracellular CFUs would decrease below that of WT as bacteria were removed from the system at a higher rate. We therefore conclude that microbiocidal vulnerability and bacterial death does not measurably account for the differences observed between swimming and non-swimming strains. These data support previous observations that intracellular killing of non-opsonized *P. aeruginosa* is <5% of available bacteria within a 45-min co-incubation time period [4]. Another alternative explanation for the current observations is that non-swimming bacterial mutants do not come into contact with phagocytes to the same degree as swimming-capable WT. To test this hypothesis we performed multiple, complementary assays. First, we performed gentamicin protection assays with WT or *motABmotCD* in the presence of surfactant in order to decrease surface tension that may inhibit contact between bacteria and phagocytes. In co-incubations performed with either the non-ionic detergents Tween80 or beta-octyl glucoside (used as a biofilm inhibitor [21]), or the artificial lung surfactant Survanta, we did not observe any increase in *motABmotCD* uptake (Figure 4B). Secondly, we tested whether forced contact between bacteria and phagocytes would overcome the phagocytic deficit of the non-swimming bacteria. To do so, we centrifuged bacteria onto BMDCs or macrophages and then subsequently assayed for phagocytosis. The degree of initial contact of WT or *motABmotCD* bacteria with the phagocytes following centrifugation was analyzed by FACS and was not different between strains (Figure 4C, inset). We observed a slight increase in CFU recovery of the non-swimming *P. aeruginosa flgK* and *motABmotCD* mutants (Figure 4C) as well as the non-swimming *V. cholerae flaA* and *motX* mutants (Figure 4D) relative to the respective swimming bacterial strains when contact was artificially initiated. However, the increased internalization did not recapitulate WT levels of phagocytosis, since non-swimming strains were still at least 10-fold more resistant to uptake as compared to their respective parental strains. These data demonstrate that phagocytic recognition is not solely dependent on contact between bacteria and phagocyte and supports a role for flagellar motion in pathogen recognition and ingestion.

### Flagellar motility enhances both the association and the uptake of bacteria by phagocytes

**Figure 5:**
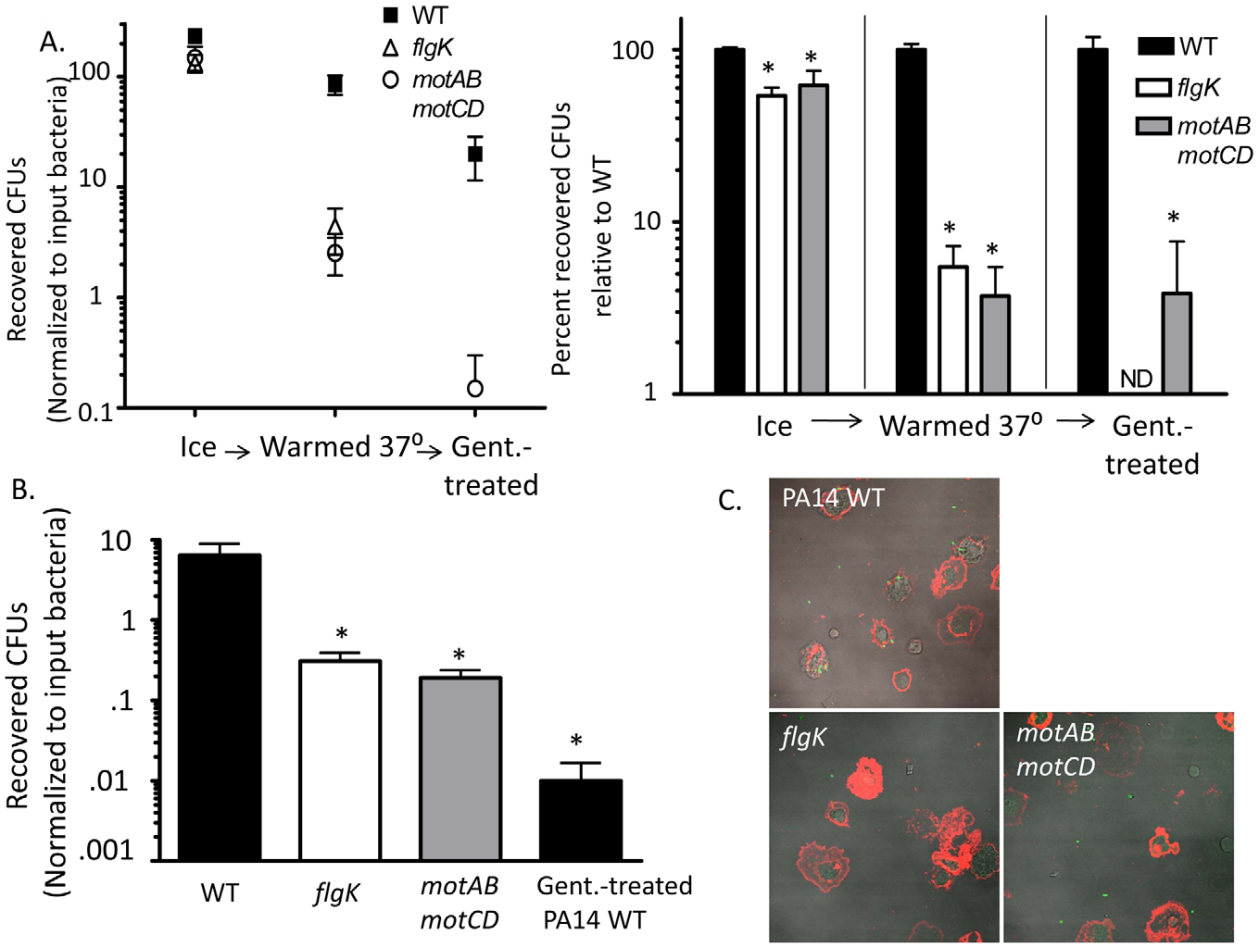
Flagellar motility enhances both the association and the uptake of bacteria by phagocytes. (A) *P. aeruginosa* PA14 WT, *flgK*, or *motABmotCD* that were incubated in parallel with adherent macrophages at 4°C exhibited similar binding (assessed by CFUs following washing and lysis of the macrophages) (left). However, the difference in relative bacterial association with macrophages dramatically changed when, following washing, the bound bacteria and cells were warmed to 37°C (middle); and this differential was even more substantial when assessed on the basis of phagocytosed (gentamicin-resistant) bacteria (right). Plots show total mean recovered CFUs accounting for input bacteria (left panel) and the number of recovered CFUs plotted relative to WT (right panel). (B) Murine peritoneal macrophages were treated with cytochalasin D prior to co-incubation with PA14 WT, *flgK,* or *motABmotCD* and were assayed for bacterial association and protection from gentamicin. (C) Fluorescence microscopy of cytochalasin D-treated macrophages co-incubated with GFP-expressing PA14 WT, GFP-expressing *flgK,* or GFP-expressing *motABmotCD* and subsequently stained with wheat germ agglutinin-conjugated Alexa647. 65x magnification. N≥5, *p<0.05.

The relative contributions of binding verses phagocytic uptake and engulfment are not well understood in non-opsonized phagocytosis. To further elucidate the individual components that promote the phagocytosis of swimming bacteria, we quantitatively assessed bacterial association with macrophages under 3 sequential conditions. We first co-incubated swimming or non-swimming *P. aeruginosa* with adherent murine macrophages at 4°C, which is permissive for binding but prevents both bacterial motility and phagocytic uptake, and then washed away non-associated bacteria and plated the cellular lysates. In parallel, we warmed cells and bacteria to 37° after the initial binding and washing at 4°C, thus initiating both bacterial movement and phagocytosis of bound bacteria, and then plated lysates directly, or treated with gentamicin and then plated. In co-incubations held at 4°C, recovered CFUs between WT, *flgK,* and *motABmotCD* were similar, as was expected since all bacteria were immobilized (Figure 5A). Of note, this also supports that it is not an unknown bacterial cell-surface ligand, with expression altered by changes in motility, that affects bacterial binding to phagocytes. However, the difference in relative bacterial association with macrophages increased dramatically when bound-bacteria and cells were warmed to 37°C, demonstrating that binding of bacteria is a necessary but insufficient component to the differential phagocytic recognition (Figure 5A). Even once associated with phagocytic cells, non-swimming *P. aeruginosa* evade uptake and, as evidenced by the progressively decreasing number of CFUs recovered after successive washes (Figure 5A left), disassociate at a higher efficiency than WT bacteria. Treatment with gentamicin demonstrated that the remaining associated bacteria, after washing, are further differentially ingested dependent on swimming-capability (Figure 5A). However, it is possible that co-incubation at 4°C distorts initial receptor-ligand interactions that nominally occur at physiological temperature. To confirm that non-swimming *P. aeruginosa* is impaired in its ability to bind innate immune cells, we pre-treated macrophages with cytochalasin D to inhibit phagocytic uptake and subsequently incubated WT or non-swimming mutants with these macrophages at 37^o^. We then washed and plated cellular lysates to quantitatively assess the bacteria that bound to the outside of the cells. In support of the previous assays, we recovered significantly fewer *flgK* and *motABmotCD* CFUs than WT (Figure 5B). Visualization of these co-incubations using GFP-expressing strains and Alexa-647-stained macrophages confirmed that bacterial association is decreased in non-swimming *P. aeruginosa* strains (Figure 5C).

### Live cell imaging of *P. aeruginosa* interactions with murine peritoneal macrophages

**Figure 6:**
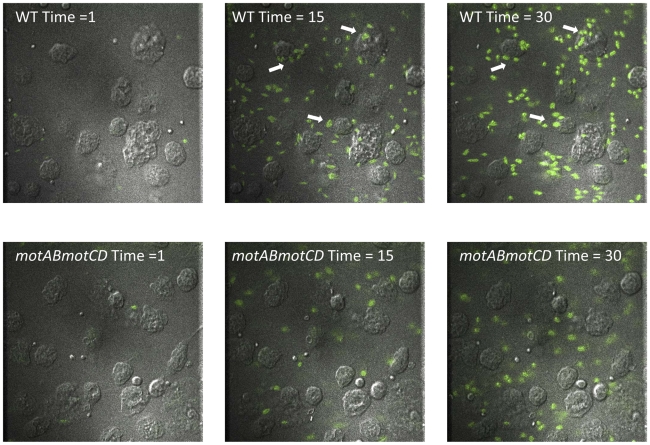
Live imaging of murine peritoneal macrophage interactions with PA14 WT or *motABmotCD* bacteria *in vitro*. Representative images of adherent macrophages treated with liquid culture of GFP-expressing PA14 WT (top) or GFP-expressing *motABmotCD* (bottom) under constant flow at Time  = 1 min, Time  = 15 min, and Time  = 30 min. Bacterial concentrations were equalized prior to imaging for comparative visualization of bacterial accumulation and retention (arrows) on phagocytes. Macrophages viewed by DIC, bacteria by fluorescence. See Videos S1 and S2.

In order to better understand and visualize how phagocytic cells bind swimming verses non-swimming bacteria we performed live cell microscopy of adherent macrophages interacting with *P. aeruginosa.* Equal concentrations of either GFP-expressing WT or GFP-expressing *motABmotCD* were flowed across adherent macrophages at a constant rate and visualized under fluorescence and DIC. WT readily accumulated on macrophage cell surfaces with prolonged associations and visible and substantial adherence events (Figure 6 top, Video S1). The *motABmotCD* mutant displayed little or no accumulation on the cells, visually flowing past macrophages with appreciably shorter adherent associations (Figure 6 bottom, Video S2). These images support the previous data which show that phagocytic evasion by non-swimming bacteria is achieved through multi-faceted resistance to binding accompanied by phagocytic unresponsiveness even with contact.

### Step-wise loss of flagellar torsion progressively increases phagocytic resistance

**Figure 7:**
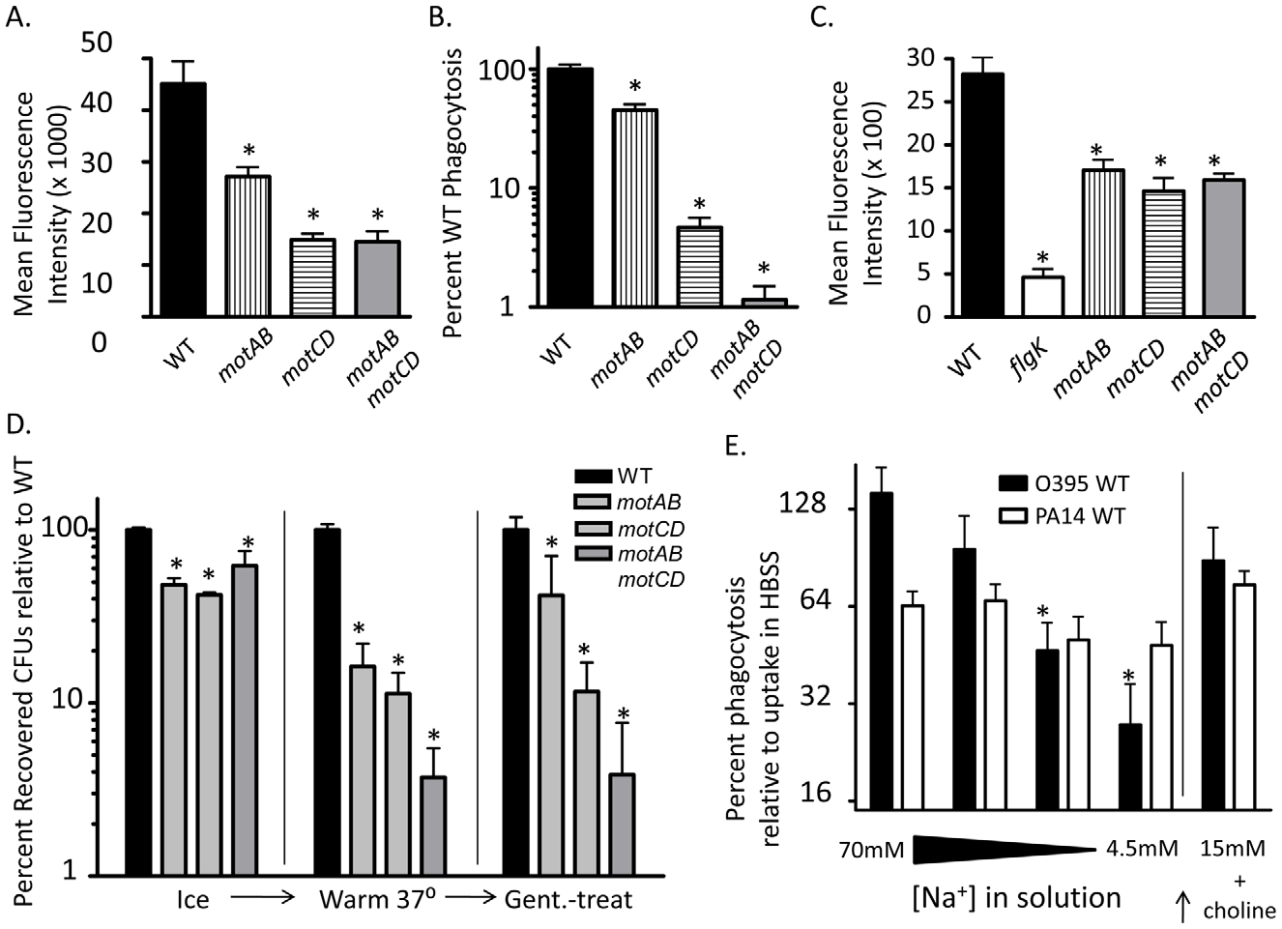
Successive loss of flagellar functionality enables stepwise increases in phagocytic resistance. (A) FACS analysis and (B) gentamicin protection assay of BMDCs co-incubated with PA14 WT, *motAB*, *motCD, or motABmotCD*. (C) FACS analysis of cytochalasin D treated BMDCs co-incubated with GFP-expressing PA14 WT, *flgK, motAB*, *motCD,* or *motABmotCD*. (D) Adherent macrophage assay of cells co-incubated with PA14 WT, *motAB, motCD, or motABmotCD* bacteria at 4°C, warmed to 37 °C, or treated with gentamicin after warming to 37 °C. (E) Gentamicin protection assay of murine peritoneal macrophages co-incubated as indicated with *P. aeruginosa* PA14 WT or *V. cholerae* O395 WT in 70 mM, 18 mM, 9 mM, or 4.5 mM NaCl buffers, or in 15 mM NaCl buffer with 135 mM choline chloride. Recovered CFUs are normalized against recovery in HBSS (138 mM NaCl). N≥5, *p<0.05.

Our data indicate that flagellar rotation confers phagocytic recognition by innate immune cells. As a formal test of this, we hypothesized that bacterial flagellar motility would be proportional to phagocytic uptake. Motility studies with *P. aeruginosa* grown in media of increasing viscosity have shown that successive genetic deletions of the partially-redundant *mot* flagellar stator complexes result in decreases in swimming capability [9], [14]. Specifically, swimming and flagellar-based motility in *P. aeruginosa* is tied to the degree of flagellar stator function, since loss of rotation from deleting *motAB* decreases flagellar-based motility below that of WT, while loss of *motCD* further decreases flagellar-based motility below that of the *motAB* mutant [9], [14], and loss of all four *mot* genes (both complexes) renders *P. aeruginosa* completely unable to swim or swarm (maximal expansion of colonies of WT, *motAB*, *motCD* and *motABmotD* in 0.6% agar were previously assessed as 29.5, 21.9, 7.3, and 6.3 mm, respectively [9], [14]). Therefore, we used isogenic *mot* mutants to test if decreases in swimming ability confer proportional increases in phagocytic evasion. Total bacterial association between GFP-expressing *motAB* and BMDCs was significantly decreased as compared to GFP-expressing WT as measured by fluorescence-activated cell sorting (FACS), while association was further decreased in GFP-expressing *motCD* and GFP-expressing *motABmotCD* (Figure 7A). To more rigorously and quantitatively assess relative phagocytosis of these mutants we returned to the gentamicin protection assay. Phagocytosis of *motAB* was slightly but significantly decreased compared to WT (Figure 7B). Further phagocytic resistance was observed in *motCD*, with non-swimming *motABmotCD* mutant being the most resistant (Figure 7B). This was not due to measurable differences in binding between the *mot* mutants, since these all bound to cytochalasin D-treated BMDCs similarly, though binding was impaired relative to GFP-expressing WT and better than GFP-expressing *flgK* (Figure 7C). Importantly, microarray analysis comparing gene expression profiles between WT, *motAB*, *motCD* and *motABmotCD* did not reveal any genetic changes that progressively correlate amongst these four strains with motility and therefore there were also no changes amongst the bacterial strains that correlated with phagocytosis (Table S2). Using methodology similar to that in Figure 5A, we next used the *mot* mutants to compare relative swimming ability with phagocytosis by adherent macrophages. Assessment of retained association and subsequent engulfment after initial binding revealed that all 3 *mot* mutants were slightly, but comparably, deficient in binding to adherent macrophages at 4°C (Figure 7D). However, upon warming of cells and bound bacteria to 37°C, followed by treatment with gentamicin, a progressive loss of association relative to WT was observed where association and engulfment of WT > *motAB* > *motCD* > *motABmotCD* (Figure 7D). This is the first evidence that the MotAB and MotCD proteins regulate phagocytic susceptibility in *P. aeruginosa* and that sequential loss of the Mot complexes confers increasing phagocytic evasion.

Since we observed increasingly dramatic phagocytic evasion phenotypes through genetic manipulation of the bacterial stator complexes, we turned to *V. cholerae* for biochemical proof-of-principle in support of our genetic evidence. Flagellar torque in *Pseudomonas* is believed to be generated through active transport of protons across the outer and inner membranes [14], [22]. In *V. cholerae*, however, flagellar torque is generated through transport of sodium ions [15], [23]. Progressively limiting the concentration of sodium in the media leads to proportional inhibition of *V. cholerae* flagellar rotation, and thus its ability to swim [23]. We performed gentamicin protection assays in serum-free buffers containing successively titrated concentrations of NaCl, while maintaining a constant osmolarity by substituting choline chloride. As expected, decreasing the availability of sodium did not significantly change the degree of uptake of WT *P. aeruginosa*, which does not depend on sodium for flagellar motility (Figure 7E). However, loss of sodium availability correlated with increased phagocytic resistance by WT *V. cholerae* (Figure 7E). Treatment of *V. cholerae* or *P. aeruginosa* alone with sodium-limited buffers did not significantly decrease recovery of colony forming units (data not shown). Importantly, reconstitution of the lowest sodium-containing buffer with 15mM NaCl, while maintaining the choline chloride concentration constant, elicited a recovery in phagocytic susceptibility of *V. cholerae*, thereby confirming that choline chloride itself is not responsible for increased phagocytic resistance or for death of the *V. cholerae* (Figure 7E). Taken together, these data indicate that the step-wise loss of flagellar torsion and swimming ability, whether through genetic deletion of the stator complex or limiting ion motive force, provides for an increasing ability to evade recognition and phagocytosis by innate immune cells.

## Discussion

In this work, we define the steps that comprise phagocytic recognition of non-opsonized bacterial pathogens and identify that changes in flagellar swimming motility can titrate the phagocytic clearance of bacteria. In support of previous results, phagocytosis is independent of flagellar assembly, since both flagellated and non-flagellated non-swimming strains are equally resistant to uptake [4]. While the majority of these studies focus on *P. aeruginosa,* we show that this immune resistance phenotype in swimming-defective strains is not specific to a single species, but represents a potentially widespread mechanism of immune evasion by Gram-negative bacteria. This phenotype is not due to motility-regulated secreted factors or compensatory changes in expression of bacterial genes. Likewise, phagocytic evasion is not due to avoidance of contact with phagocytic cells. Instead, as shown with *P. aeruginosa*, non-swimming strains avoid phagocytic recognition by disassociation after initial contact and remain resistant to phagocytosis even after being bound at the cell surface.

In comparing resistance across multiple bacterial species, it is interesting that non-swimming *V. cholerae* mimicked *P. aeruginosa* in terms of scope and magnitude of phagocytic resistance, while the degree of non-swimming *E. coli* resistance was much less dramatic. Since loss of motility in the *V. cholerae* and *E. coli* isogenic mutants was confirmed by swimming motility assays in 0.3% agar (data not shown), it is not immediately clear why swimming deficiency in *E. coli* conferred resistance to a lesser degree. Of note, both *P. aeruginosa* and *V. cholerae* have a single, polar, monotrichous flagellum under standard conditions, whereas *E. coli* swim via multiple peritrichous flagella [9], [12], [13]. However, non-flagellated *flgK* and *fliC* strains of *E. coli* were not significantly different than the flagellated, non-swimming *motA* mutant in terms of phagocytic uptake. It is therefore unlikely that multiple flagella are causative of the discrepancy; more plausible is that the recognition of alternative structures, specific to *E. coli*, are able to partially compensate for the resistance phenotype of non-swimming strains. The small decrease in uptake observed with a mucoid strain of swimming-capable *P. aeruginosa*, as well as the trending decrease in recovered *V. cholerae* CFUs when TCP is eliminated, suggest that such compensatory mechanisms are feasible. Even so, the significant drop in phagocytic susceptibility when *E. coli* loses flagellar motility, independent of flagellar assembly, supports our hypothesis of a widespread mechanism utilized by innate immune cells for phagocytosis of motile, non-opsonized pathogens. Moreover, we demonstrate that this response to motility is shared amongst phagocytic cell types, anatomical locations, and cells of human or mouse origin (Figure 1 and [4]).

To delineate the phagocytic process, we examined bacterial engulfment in a step-wise manner. Our data indicate that for an uptake event to occur, contact must be made between the pathogen and the phagocyte, followed by adhesion and recognition, which culminates in a stimulus to ingest. In assessing *P. aeruginosa* binding and association with cytochalasin D-treated BMDCs, both the non-flagellated *flgK* mutant and the flagellated *mot* mutants were modestly but significantly decreased in binding to BMDCs compared to WT, though the degree of *flgK* association was well below that of all three *mot* mutants, which were not different from each other. This is of interest since multiple reports have identified bacterial flagella as potent adhesins in physiological systems [24]–[26]. Taken together, these data point to the flagellum in *P. aeruginosa* as indeed having adhesive properties, but calls into question the role of flagella as direct ligands for phagocytosis since all non-swimming mutants, regardless of flagellar expression, were indistinguishable in phagocytic assays. Furthermore, artificial enforcement of contact between non-swimming *P. aeruginosa* or *V. cholerae* and phagocytes did not recapitulate WT levels of uptake. In the experiments where pathogen-host cell contact was induced, and therefore initially equal between strains, we recovered significantly fewer CFUs using non-swimming strains relative to controls and the presence of the flagellum did not change phagocytic uptake among strains that were non-swimming. Therefore, the presence of a non-rotating flagellum can only partially recover WT levels of binding to phagocytic cells, and contact alone, regardless of flagellar assembly, is necessary but insufficient for full phagocytic activation. Importantly, among those bacteria that bound to the cell-surface, we discovered that their subsequent phagocytic fate is dependent on their swimming capability. Overall dissociation after contact, and therefore also evasion of engulfment, was increased in non-swimming *P. aeruginosa* compared to WT. These data support an overall model where loss of flagellar rotation enables the evasion of both phagocyte binding and, importantly, recognition and response after initial contact.

The evidence that the flagellum itself is not contributing to phagocytic susceptibility, but that flagellar rotation is, raises the question of how the cells preferentially recognize swimming bacteria. We previously demonstrated that loss of the MyD88 adaptor protein did not alter phagocytic uptake of *P. aeruginosa* [4]. Therefore, none of the MyD88-dependent toll-like receptors (TLRs), specifically TLR5 which recognizes bacterial flagellin, are required for phagocytosis of *P. aeruginosa*. The two likely possibilities, not mutually exclusive, are that bacterial motility alters the expression of an unknown bacterially-produced factor or ligand that alters the ability of the phagocyte to recognize or ingest the bacteria; or that the phagocyte can sense the motility and that this drives the phagocytic event. Our data demonstrate that it is unlikely that phagocytic cells are sensing a motility co-regulated secreted molecule or extracellular ligand, since the mixed-culture assay did not indicate that WT could confer phagocytic susceptibility to the *motABmotCD* mutant *in trans*, nor could *motABmotCD* confer phagocytic resistance to WT. This inability to physiologically complement the phagocytic phenotype suggests that the extracellular environment of cells co-incubated with WT is not different than when cells are co-incubated with *motABmotCD.* In support of the complementation data, microarray analysis of bacterial gene expression when the *mot* complexes were successively deleted from WT indicate that no known immunogenic effectors are significantly altered. Most importantly, there was no significant change in gene expression pattern that correlated with successive deletion of the *mot* complexes and therefore no compensatory bacterial genetic changes that correlated with phagocytic susceptibility. Those genes that do change expression more than 2-fold in response to deletion of *mot* genes are likely bystander effects, as we could not identify phagocytic or motility phenotypes in corresponding transposon mutants. Thus, we believe it is unlikely that phagocytic evasion by *motABmotCD* is due to indirect effects of *mot* gene deletion. The alternative explanation is that leukocytes possess a mechanism that recognizes and responds to flagellar torsion as a phagocytic initiation signal. We hypothesized that, if this were the case, then step-wise decreases in flagellar torsion would result in proportional increases in phagocytic resistance. Deleting MotAB from the stator complex of *P. aeruginosa* results in decreased swimming speed, as it partially contributes to flagellar torque generation [9], [14]. Our data show that this decrease in swimming capability confers a small but significant degree of resistance to phagocytosis. Further loss of flagellar motility, due to deletion of MotCD, which is a larger contributor to stator functionality than MotAB [9], [14], conferred a greater degree of phagocytic resistance. Complete loss of flagellar function, the phenotypic result of deletion of all four *mot* genes [9], [14], conferred the greatest degree of phagocytic resistance, equal to that of non-flagellated mutants. Once bacteria are cell-bound, subsequent dissociation and phagocytic evasion follow the same pattern, with resistance in *motABmotCD* > *motCD* > *motAB* > WT. This is the first demonstration of titrated phagocytic resistance in *P. aeruginosa* being regulated through *mot* gene function, and fits an infection model where the sensory mechanisms of the innate immune system provide a selective pressure for *P. aeruginosa* to down-regulate flagellar motility. Since the partial loss of the stator does not completely inhibit swimming capability, selective pressure to lose stator functionality would not necessarily impede *P. aeruginosa* colonization, but provide increasing degrees of resistance to phagocytic recognition. Indeed, the partial redundancy in the *P. aeruginosa* stator proteins may have evolved to provide such an advantage during infection.

While a functional stator complex is required for flagellar rotation, additional requirements, such as the electro-chemical gradient that provides rotational force, are necessary for full flagellar motility [23]. Limiting the availability of ions required for flagellar rotation can selectively impede flagellar motility [23]. We found that progressively decreasing sodium availability to *V. cholerae*, which depends specifically on sodium ions for flagellar rotation, conferred step-wise increases in phagocytic resistance, analogous to our observations with the *P. aeruginosa* genetic mutants. Limiting sodium availability to *P. aeruginosa* did not alter phagocytic susceptibility, which fits with *P. aeruginosa* use of a proton motive force and not sodium for flagellar motility [14], [22]. These data are in agreement with our results using genetically modified bacteria, indicating that loss of flagellar motility, regardless of the means, confers resistance to phagocytic uptake.

While the down-regulation of flagellar motility in *P. aeruginosa* isolates from persistent infections has been previously documented [5]–[7], these results provide an explanation for the observed loss of motility in clinical strains recovered from CF patients over the course of chronic infection, but which is not limited to just *P. aeruginosa*. Consistent with a pleiotropic mechano-sensory system, both non-swimming *V. cholerae* and *E. coli* also demonstrate phagocytic resistance. We believe it is therefore likely that innate immune cells are able sense bacterial motility, possibly through membrane depression or activation of an unknown tension receptor(s), and that this mechanical perturbation, analogous to a “fish on a hook”, provides the necessary sensory stimulant for the cell to “set the hook” and initiate phagocytic uptake. Examples of cellular mechano-sensory systems exist in other physiological systems, such as cellular stretch detection in muscle sarcoma cells [27] and shear-enhanced adhesive catch bonds in rolling leukocytes [28], but to date no reports have identified such a mechanism contributing to pathogen recognition. Since flagellar motility is a necessary virulence factor for many pathogens to effectively colonize a host [29]–[32], it makes evolutionary sense that the innate immune system, as a first line of defense, would develop strategies to exploit this phenotype. Concomitantly, loss of flagellar motility in isolates taken from established infections corresponds to selective pressure to bypass this immune strategy.

In conclusion, in this work we demonstrate that bacterial flagellar rotation is recognized as a phagocytic activator by innate immune cells. We show that this mechanism responds to at least three different species of bacteria, *P. aeruginosa, V. cholerae,* and *E. coli*, and thus likely represents a common and widespread immune strategy for bacterial recognition by direct sensing of flagellar torsion. In the *P. aeruginosa* model, swimming-deficient strains avoid phagocytic uptake through a combinatorial strategy of limiting prolonged association after initial contact with phagocytic cells and not eliciting uptake when bound to the cell surface. We show for the first time that phagocytic recognition is directly proportional to *mot* gene function as it relates to phenotypic flagellar torsion. These results provide a basis for the reported observations of non-motility in clinical strains isolated from established infections, and provide evidence of a novel strategy utilized by the innate immune system to fight bacterial infection.

## Materials and Methods

### Ethics statement

This study was carried out in strict accordance with the recommendations in the Guide for the Care and Use of Laboratory Animals of the National Institutes of Health. The protocol was approved by the Dartmouth IACUC Committee (Permit Number: A3259-01). No surgery was performed, and all efforts were made to minimize suffering.

### Mice and cells

Bone marrow-derived dendritic cells (BMDCs) were cultured from C57BL/6 WT mice obtained from NCI using a modification of Inaba *et al.* as previously described [33]. For isolation of murine macrophages, naïve C57BL/6 mice were injected i.p. with 1 ml of 4% thioglycolate and subsequently sacrificed 4 days later. The peritoneal cavity was lavaged with 6 ml of serum-free Hank's Balanced Salt Solution (HBSS). The lavage fluid was centrifuged and pelleted cells were washed twice in serum-free HBSS before being resuspended in 2 ml serum-free HBSS. For these studies, the *Pseudomonas aeruginosa* strain FRD1 is a mucoid clinical isolate, while the non-mucoid clinical isolate PA14, *Vibrio cholerae* strain O395 (generously provided by Dr. Ron Taylor, Dartmouth), and *Escherichia coli* strain K12 (obtained from Yale CGSC) are the parental bacterial strains and wild type controls for all of the respective mutants studied.

### Bacterial binding assays and FACS-based bacterial association

Bacterial strains expressing green-fluorescence protein (GFP) were generated by transformation of the indicated strains with a multi-copy plasmid (pSMC21 Amp^r^ Kan^r^ Carb^r^ GFP*^+^*) that constitutively expresses GFP under control of a derivative of the P_tac_ promoter [34], [35]. FACS-based bacterial association was assayed as a modified version previous protocols [4]. Briefly, 2.5×10^5^ C57BL/6 BMDCs or macrophages were incubated with the indicated non-transformed or GFP-expressing bacterial strains at an MOI of ∼10 in serum-free HBSS for 45 minutes at 37°C or 4°C as indicated in the text. Cells were washed in phosphate-buffered saline (PBS) and mean fluorescence intensity of the phagocyte populations were assessed and graphed to obtain relative efficiency of cellular association with the indicated bacterial strains. For bacterial binding assays, BMDCs or macrophages were pre-incubated in 10 uM cytochalasin D (Sigma) in serum-free HBSS for 60 minutes at 37°C. Co-incubation between phagocytes and the indicated bacterial strains took place in the presence of 10 uM cytochalasin D in serum-free HBSS or in HBSS alone for 45 minutes at 37°C. Cells were subsequently washed in serum-free HBSS or PBS and then analyzed by plating cellular lysates and counting recovered CFUs, or by FACS for the acquisition of fluorescence as a function of GFP^+^ bacterial association.

### Gentamicin protection assays

Phagocytosis of live bacteria was performed as a modified version of published protocols [36] and as previously described [4]. Briefly, overnight cultures of *P. aeruginosa, V. cholerae,* or *E. coli* were washed and resuspended in serum-free HBSS or the indicated buffer and bacteria concentrations were determined. 2.5×10^5^ BMDCs or the indicated cell type were incubated with bacteria at an MOI of ∼10 for 45 minutes at 37°C, followed by incubation in 100 µg/ml gentamicin for 20 minutes at 37°C. Recovered CFUs are normalized to input bacteria to account for variability in initial strain concentration. Where indicated, recovered CFUs are presented as a percent of the isogenic WT to compare relative degrees of phagocytosis. In experiments utilizing sodium-limited buffers, solutions were made with 0.9 mM CaCl, 4 mM KCl, 0.5 mM MgCl, 5 mM HEPES and the indicated amount of NaCl and reconstituted with choline chloride for a combined concentration of 140 mM (pH 7.5). For phagocytic threshold experiments, the concentration of non-swimming *P. aeruginosa* was successively increased 10-fold relative to the concentration of WT. For forced-contact experiments, BMDCs or macrophages were centrifuged for 5 min at 400 g. Bacteria were then layered onto pelleted cells followed by centrifugation at 4°C for 10 min at 715 g. An equal degree of cell-to-bacteria contact after centrifugation in swimming verses non-swimming strains was verified by immediately fixing cells and GFP-transformed bacteria with 4% paraformeldahyde and measuring the accumulated cellular GFP signal via FACS. For bacteria-phagocyte cell surface tension experiments, gentamicin protection assays were performed in the presence of the indicated concentrations of surfactant.

### Microarray

RNA from *P. aeruginosa* strains was prepared with TRI Reagent (Sigma) followed by the RNeasy kit (Qiagen), following manufacturer's instructions. Microarray analysis was performed on a *Pseudomonas aeruginosa* PA01 gene chip using raw oligonucleotide probes generated from wild-type PA14, the *motAB* mutant, the *motCD* mutant, or the *motABmotCD* mutant. Each sample was analyzed in triplicate (N = 3), and summarized in one probe intensity by the Vermont Genetics Network Microarray Facility using Affymetrix GCOS software. Data analysis was performed using R [37] / BioConductor tools [38], [39]. Probe set sample matrix expression statistics were calculated using the Robust Multichip Average (RMA) method of Speed and coworkers [40], [41], implemented in the aroma.affymetrix package of Bengtsson [42]. Quality statistics were calculated using the Simpleaffy [43] and AffyQCReport packages [44]. The linear mixed effects model was fit using the lme4 package [45].

### Microscopy

For static imaging, BMDCs were washed twice in 400 uL of serum-free HBSS prior to a 10 minute cytospin onto glass slides at 89.5 g. Alternatively, primary macrophages were allowed to adhere to glass slides for 1 hour at 37°C. Cells were co-incubated with GFP-expressing *P. aeruginosa* or GFP-expressing *V. cholerae* strains as indicated for 45 minutes at 37°C at a MOI∼10. Cells were stained with Alexa647-labeled wheat germ agglutinin (Molecular Probes) to delineate the cell surfaces. Cells were visualized via fluorescence microscopy on a Zeiss LSM510 Meta microscope using a 40X or 63X lens, followed by image analysis with LSM5 Image Browser software. For live cell imaging, GFP-expressing *P. aeruginosa* in phosphate buffer saline (PBS) were flowed over a monolayer of adherent macrophages at 50 mL/h for 20 min at an MOI∼10, followed by fresh media for 10 min. Bacterial accumulation was monitored at 5 sec intervals at 60X magnification using fluorescence and DIC. Imaging was performed using a Nikon TE2000 swept field confocal microscope with 0.17 mm Delta TPG dishes and analysis was performed with NIS-Elements viewing software.

### Adherent macrophage assays

Macrophages were allowed to adhere to the bottom of 24- or 48-well plates in serum-free HBSS for 60 min at 37°C. Adherent cells were washed twice in serum-free HBSS followed by co-incubation with *P. aeruginosa* strains at 4°C for 30 min. Non-associated bacteria were removed by washing with serum-free HBSS. Cell-associated bacteria were quantified by lysing cells in 0.1% Triton-X 100, plating lysate on LB media for >12 hours at 37°C, and counting recovered CFUs. Alternatively, following co-incubation at 4°C and removal of non-associated bacteria, cells and associated *P. aeruginosa* were warmed to 37°C for 30 min and quantified as above or warmed for 30 min, washed with serum-free HBSS, treated with 100 ug/mL gentamicin, and then quantified as above.

### Bacterial viability assays

*P. aeruginosa* or *V. cholerae* cultures were grown to mid-log phase in LB broth and diluted to O.D. 600<0.1 in serum-free HBSS. To measure susceptibility to gentamicin, samples were treated with 100 ug/mL for 15 minutes at 37°C and then directly plated on LB agar or, alternatively, untreated samples were further diluted 1∶200 (*P.a.*) or 1∶555 (*V.c.*) and plated on LB agar and resultant CFUs were counted. To measure bacterial replication and death in HBSS, bacterial cultures were prepared as above and then plated directly, or incubated at 37°C for 60 min and quantified as above.

## Supporting Information

Figure S1 **Loss of the flagellum or flagellar motility does not confer changes in bacterial viability or gentamicin susceptibility.** Recovered CFUs of (A) *P. aeruginosa* or (B) *V. cholerae* before and after 15 minute incubation with 100 ug/mL gentamicin in serum-free HBSS at 37°C. (C) Recovered CFUs of *P. aeruginosa* WT, *flgK,* or *motABmotCD* or (D) *V. cholerae* WT, *flaA, or motX* before and after 60 minute incubation at 37°C in serum-free Hank's balanced salt solution (HBSS).
(TIF)

Table S1 **Change in gene expression of bacterial immunogens with loss of** ***motABmotCD*** **.** Selected genes detected in bacterial microarray that correspond to known or proposed immunogenic molecule expression by *P. aeruginosa*, compared to gene expression with loss of *motABmotCD.*
(XLSX)

Table S2 ***P. aeruginosa*** **genes detected in bacterial microarray.** List of genes identified in bacterial microarray analyses with comparative expression levels and statistical analysis relative to loss of *motAB*, *motCD*, or *motABmotCD*.
(XLSX)

Video S1 **Live cell microscopy of macrophage interactions with PA14** ***Pseudomonas aeruginosa*** **.** Adherent macrophages (viewed in DIC) were treated with liquid culture of GFP-expressing PA14 WT under constant flow. Images are recorded every 5 seconds over a 30 minute time period and compressed for playback. Bacterial concentrations were equalized prior to imaging for comparative visualization of bacterial accumulation and retention on phagocytes.
(WMV)

Video S2 **Live cell microscopy of macrophage interactions with** ***motABmotCD*** ***Pseudomonas aeruginosa*** **.** Using the same methodology and bacterial concentrations as in Video S1, the accumulation of GFP-expressing *motABmotCD* PA14 bacteria by adherent macrophages was visualized.
(WMV)
