# Supplementary figures and images for: Step-Wise Loss of Bacterial Flagellar Torsion Confers Progressive Phagocytic Evasion
Source: PLoS Pathog. 2011 Sep 15;7(9):e1002253. doi: 10.1371/journal.ppat.1002253 (PMC3174259; doi:10.1371/journal.ppat.1002253)

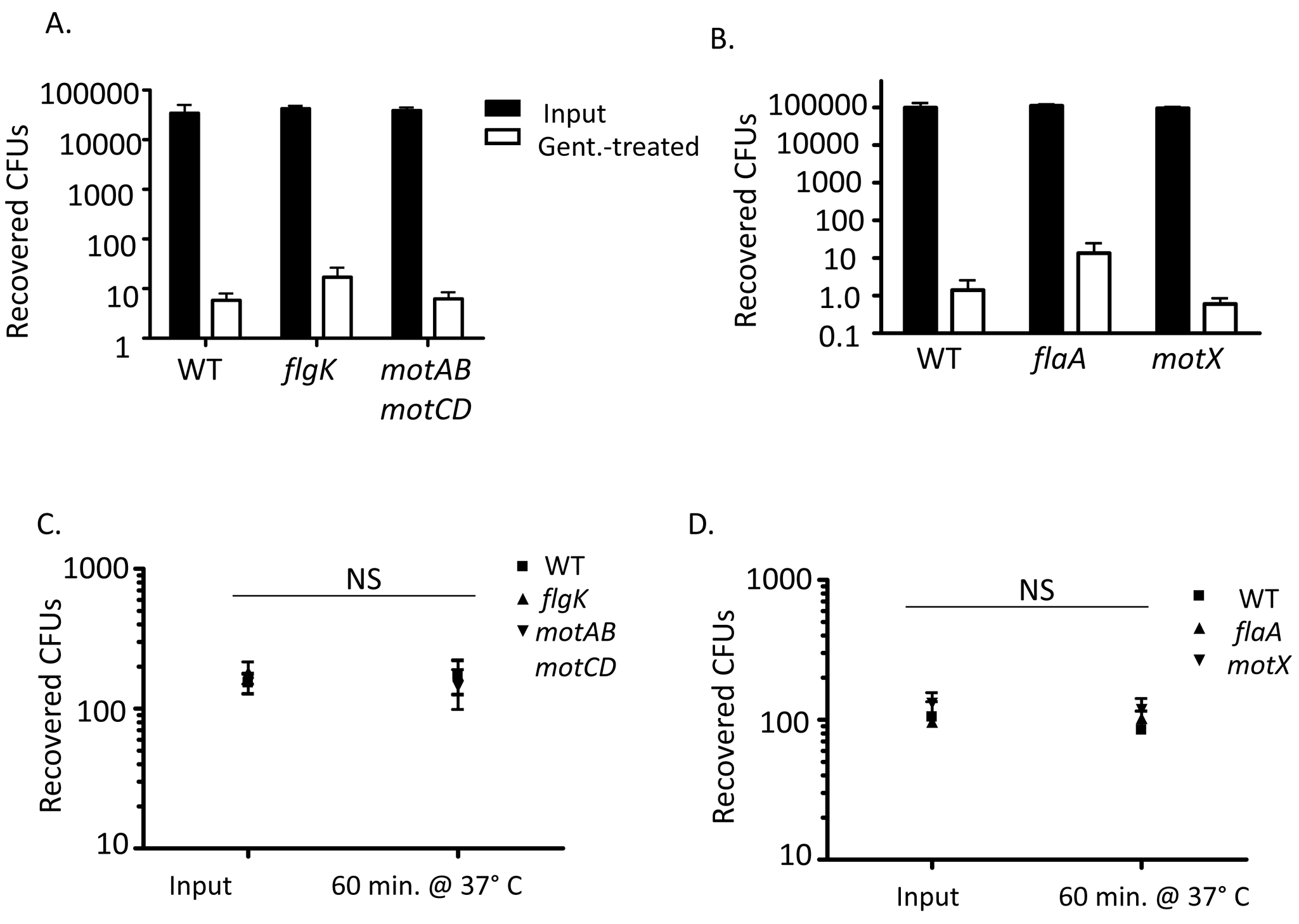

Supplement: Figure S1 — Loss of the flagellum or flagellar motility does not confer changes in bacterial viability or gentamicin susceptibility. Recovered CFUs of (A) P. aeruginosa or (B) V. cholerae before and after 15 minute incubation with 100 ug/mL gentamicin in serum-free HBSS at 37°C. (C) Recovered CFUs of P. aeruginosa WT, flgK, or motABmotCD or (D) V. cholerae WT, flaA, or motX before and after 60 minute incubation at 37°C in serum-free Hank's balanced salt solution (HBSS). (TIF) [file ppat.1002253.s001.tif]
